# Recent Progress in Germplasm Evaluation and Gene Mapping to Enable Breeding of Drought-Tolerant Wheat

**DOI:** 10.3389/fpls.2020.01149

**Published:** 2020-08-04

**Authors:** Kamal Khadka, Manish N. Raizada, Alireza Navabi

**Affiliations:** Department of Plant Agriculture, University of Guelph, Guelph, ON, Canada

**Keywords:** drought tolerance, genetic resources, landraces, quantitative trait loci mapping, wheat, climate change

## Abstract

There is a need to increase wheat productivity to meet the food demands of the ever-growing human population. However, accelerated development of high yielding varieties is hindered by drought, which is worsening due to climate change. In this context, germplasm diversity is central to the development of drought-tolerant wheat. Extensive collections of these genetic resources are conserved in national and international genebanks. In addition to phenotypic assessments, the use of advanced molecular techniques (e.g., genotype by sequencing) to identify quantitative trait loci (QTLs) for drought tolerance related traits is useful for genome- and marker-assisted selection based approaches. Therefore, to assist wheat breeders at a critical time, we searched the recent peer-reviewed literature (2011-current), first, to identify wheat germplasm observed to be useful genetic sources for drought tolerance, and second, to report QTLs associated with drought tolerance. Though many breeders limit the parents used in breeding programs to a familiar core collection, the results of this review show that larger germplasm collections have been sources of useful genes for drought tolerance in wheat. The review also demonstrates that QTLs for drought tolerance in wheat are associated with diverse physio-morphological traits, at different growth stages. Here, we also briefly discuss the potential of genome engineering/editing to improve drought tolerance in wheat. The use of CRISPR-Cas9 and other gene-editing technologies can be used to fine-tune the expression of genes controlling drought adaptive traits, while high throughput phenotyping (HTP) techniques can potentially accelerate the selection process. These efforts are empowered by wheat researcher consortia.

## Introduction

Bread wheat (*Triticum aestivum* L.) is one of the world’s major cereal crops with global production of 756.7 million tons in 2017 (FAO, 2018). The world’s population is expected to exceed 9 billion by 2050, requiring at least a 60% increase in wheat yield (United Nations, 2019). An increase in wheat yield from the current level of 1% per year to at least 1.6% is deemed necessary to address this challenge (GCARD, 2012). This is further challenged by projected unpredictable rainfall patterns associated with climate change which are expected to lead to more drought events (IPCC, 2013). However, the degree of impact on final yield depends on the growth stage and the intensity and duration of stress events (Daryanto et al., 2016; Sarto et al., 2017).

Like many other crops, drought affects wheat at all growth stages (HongBo et al., 2005; Saeidi et al., 2015; Saeidi and Abdoli, 2015; Wang et al., 2015; Sarto et al., 2017; Ding et al., 2018). Some of the growth stage-specific physio-morphological traits associated with drought tolerance in wheat include: early vigor (Rebetzke et al., 1999), coleoptile length (Rebetzke et al., 2007), leaf chlorophyll content (Khayatnezhad et al., 2011; Kira et al., 2015; Ramya et al., 2016), glaucousness (waxiness) for photo-protection (Merah et al., 2000; Bi et al., 2017), leaf rolling (Kadioglu and Terzi, 2007), carbon isotope discrimination (Kumar and Singh, 2009), flag leaf senescence (Verma et al., 2004; Hafsi et al., 2013) and plant height (Su et al., 2019). Also, root system architecture (RSA) traits are fundamental targets for breeding drought-tolerant wheat varieties (Lopes and Reynolds, 2010). A breeding program that selects for these physio-morphological traits has the potential to contribute to drought stress tolerance in wheat, as recently reviewed (Khadka et al., 2020).

In general, breeding can be accelerated by exploiting the diversity of genetic resources as sources of alleles that enhance desirable traits. More than 800,000 wheat accessions, including local landraces and synthetics, are conserved in genebanks globally (FAO, 1998). A large proportion of these accessions are accessible to wheat breeders. Recent advancements in genomics (e.g., genotype by sequencing, see below) have enabled exploration of this genetic diversity, leading to the discovery of markers and associated quantitative trait loci (QTLs) that can be utilized in marker assisted selection and genomic selection to accelerate variety development (Huang and Han, 2014). Wheat has a large and complex genome (Marcussen et al., 2014; Shi and Ling, 2017; Uauy, 2017; Abrouk et al., 2018). Very recently, however, an annotated reference whole genome sequence for bread wheat was released, which described 107,891 high-confidence level genes (IWGSC, 2018). This release has provided a significant opportunity to use genetic resources for exploring the wheat genome, and selecting alleles that encode desirable physio-morphological traits associated with drought tolerance.

There have been excellent recent reviews on the progress in breeding wheat and other cereals for tolerance to abiotic stress including drought (Mohammadi, 2018; Choudhary et al., 2019; Gupta et al., 2020). However, a unique feature of this current paper is that it reviews the different genetic resources that have been or can be exploited to accelerate the breeding of wheat for drought tolerance. The paper comprehensively updates recent progress (2011–2020) on the discovery of QTLs that promote drought tolerance in wheat, expands the list of associated physio-morphological traits and provides helpful details to breeders (e.g., QTL source populations). The QTL section is now the most up-to-date review on the topic. Furthermore, this paper also presents very recent advances in genetic engineering including the CRISPR/Cas9 genome editing system that has been used to target genes conferring drought tolerance.

## Utilization of Genetic Variation From Diverse Sources

Use of diverse germplasm is key to the development of drought-tolerant wheat varieties. As noted in **Table 1**, *T. aestivum* landraces are one of the major groups of genetic resources valuable for breeding drought-tolerant wheat (Mwadzingeni et al., 2017). They have complex morphological diversity and are mostly grown in low input environments (Padulosi et al., 2012) which make them more adapted to stress (Padulosi et al., 2012; Lopes et al., 2015a). For example, a group of Creole wheat landraces (the landraces introduced to Mexico from Europe) showed better adaptation to different abiotic stresses, including drought, due to the presence of rare but beneficial alleles (Vikram et al., 2016). Similarly, the Japanese landrace “Aka Komugi” is one of the sources of the dwarfing *Rht8c* allele (Lopes et al., 2015a; Grover et al., 2018) that contributes to breeding drought tolerant wheat. This is because *Rht8c* promotes a higher-yielding semi-dwarf phenotype, but does not reduce the length of the coleoptile and thus permits deep sowing (Lopes et al., 2015a). Furthermore, in a study that evaluated 21 genotypes, nine wheat landraces exhibited drought tolerance based on a stress susceptibility index (SSI) (Sareen et al., 2014).

**Table 1:** Recent peer reviewed reports (2011-current) of germplasm evaluation for assessing drought tolerance in wheat.

#	Collaborating institutions	Experiment/evaluation	Germplasm type	Number of genotypes	Drought treatment	Results	Reference
1	Bread Wheat Breeding Program, CIMMYT	Evaluation of high yielding genotypes using data from 740 international Semi-Arid Wheat Yield Trials (SAWYT) conducted in 66 countries from 2002–2003 to 2013–2014 was performed to determine genetic gain for grain yield.	The SAWYT included advanced breeding lines including SHW derivatives and local checks.	50 entries in each SAWYT	Experiments conducted using local management practices. After 2 year testing under optimum irrigation and 1–2 years of testing under drought and heat stress, the elite lines were selected.	Results showed broader use of genotypes Pastor, Baviacora 92, and synthetic hexaploid derivatives to develop stable and drought-tolerant wheat lines.	(Crespo-Herrera et al., 2018)
2	University of KwaZulu-Natal (UKZN), Pietermaritzburg, South Africa Agricultural Research Council-Small Grain Institute, Bethlehem, South Africa University of South Africa, Pretoria, South Africa	A panel of wheat genotypes were tested under greenhouse and field conditions during 2014/15 and 2015/16.	Advanced CIMMYT lines and local checks	96 lines were tested (including 88 lines from CIMMYT’s heat and drought nurseries and 8 local checks).	Drought stress treatments were applied after heading until maturity by withholding irrigation to 35% of field capacity	Twelve CIMMYT lines were selected as drought-tolerant after the evaluation.	(Mwadzingeni et al., 2016b)
3	Razi University, Kermanshah, Iran	A set of wheat genotypes were evaluated under rain-fed and normal irrigated conditions in 2010–11 season using 13 drought tolerance indices.	Landraces	12	The drought stress was applied by supplying no irrigation, while the non-stressed treatments were provided with irrigation.	The landraces WC-4953S, WC-47572, and WC-47574 were identified as drought-tolerant.	(Farshadfar et al., 2012)
4	Kerman University, Iran	The wheat genotypes previously reported as drought-tolerant, were evaluated for drought tolerance using nine different drought indices under normal and water stressed conditions for two seasons in 2009–10 and 2010–11.	Landraces and modern cultivars	40	Cyclic drought stress was applied in a glasshouse experiment. The field experiments were conducted under fully irrigated and rainfed conditions.	Landrace Mahdavi was identified as the most drought-tolerant genotype.	(Abdolshahi et al., 2013)
5	Indian Institute of Wheat and Barley Research, Karnal, India	The wheat genotypes representing major wheat growing zones in India were tested for two seasons under different water regimes using the three marked water stress indices.	Modern cultivars	15	Normal (5 irrigations) and restricted irrigation treatments were applied to assess drought tolerance.	Three genotypes, NI-5439, WH-1021, and HD-2733, were identified as the most drought-tolerant cultivars.	(Meena et al., 2015)
6	University of Reading, United Kingdom	A set of wheat genotypes were assessed for drought tolerance in an ambient glasshouse environment using different drought indices.	Modern cultivars and a local check	6	Three watering regimes (100%, 35%, and 25% capacity) were used to test for drought tolerance.	Hashim-8 was identified as the superior variety for drought tolerance.	(Khakwani et al., 2011)
7	Mansoura University, Mansoura, Egypt	The wheat genotypes were evaluated for the effect of two levels of osmotic stress at seedling stage.	Modern cultivars (released varieties from 1999 to 2011)	10	The treatments were normal irrigation until 45 days, and irrigation withheld for 21 days in the stress treatment.	The results showed a negative effect of the stress on morphological seedling traits. However, variation was observed among the genotypes. Sids 13, one of the tested genotypes, was the most drought-tolerant, while Shandawel 1 was observed to be the most sensitive genotype.	(Mickky and Aldesuquy, 2017)
8	Mansoura University, Mansoura, Egypt University of Debrecen, Hungary	Hungarian wheat landraces were selected and tested for different physiological traits at the seedling stage.	Landraces	7	Five levels of water stress were applied on seedlings (0%, 6%, 12%, 18%, and 24%) using PEG-6000.	Two landraces, Leweucei and Mateteleki, were found to be more drought-tolerant based on different drought tolerance related parameters such as higher relative water content (RWC), tolerance index (TI) and activities associated with α and β-amylases.	(Abido and Zsombik, 2018)
9	Nanjing Agricultural University, PR China	One drought-tolerant (Luhan-7) and one drought sensitive (Yangmai-16) variety were evaluated to assess improved tolerance to water stress during post-anthesis growth phase as a result of pre-drought priming at different stages during the vegetative growth phase.	Modern winter wheat cultivars	2	Drought priming was done at tillering and jointing stages with moderate stress (55–60% of field capacity). Severe stress was applied at 35–40% field capacity, seven days after anthesis.	Results showed positive effect of drought priming on both varieties when they were exposed to post-anthesis drought. However, the drought-tolerant genotype showed greater response to priming, while the growth stage of priming also contributed to drought tolerance to some extent.	(Abid et al., 2016)
10	Cereal Research Non-Profit Ltd., Hungary Snowy River Seeds Pty Ltd., Australia	Wheat genotypes were phenotyped for different root and shoot traits under drought in a glasshouse along with one drought-tolerant and one drought susceptible check.	Modern cultivars (released varieties)	29	The well-watered pots were irrigated to 60% soil water capacity, while the drought stress pots were irrigated to 20% soil water capacity.	Based on final grain yield, three varieties were identified as drought-tolerant.	(Nagy et al., 2018)
11	Punjab Agricultural University, Ludhiana, India	A group of genotypes consisting of 57 *Aegilops tauschii* accessions and 26 *Triticum dicoccoides* accessions were used to assess adaptive plasticity induced by water stress for different morpho-physiological characters such as root-shoot development, induction of proline and cell membrane injury.	Wild relatives	83	PEG based water stress was imposed at different concentrations (10, 15, 20, and 25%).	Some of the *Ae. tauschii* accessions such as 9816, 1409, and 14128, and *T. dicoccoides* accessions 5259 and 7130, exhibited significantly higher adaptive plasticity for water stress.	(Suneja et al., 2019)
12	The University of Western Australia, Perth, Australia The Bangladesh Agricultural Research Institute, Gazipur, Bangladesh	Near isogenic lines (NILs) derived from the cross between C306 and Dharwar Dry, two varieties with spring growth habit, were evaluated targeting a QTL on chromosome 4BS that confers drought tolerance.	Putative NILs pairs	10	A glasshouse experiment was performed by maintaining 80% field capacity of moisture in the control treatment, and no water was supplied for 7 days after anthesis in the stress treatment.	Results showed that NILs having C306 background out-performed the NILs with the Dharwar Dry background, while one isoline qDSI.4B.1-10(-) that carried an allele from Dharwar Dry showed superiority over the corresponding isoline with the C306 background.	(Mia et al., 2019)
13	Northwest Agricultural and Forestry University, Yangling, China	Wheat genotypes representing important wheat growing areas in China was evaluated in irrigated and water-limited environments using rainout shelters for 14 traits, including morpho-physiological and yield traits to distinguish genotypes tolerant to drought stress.	A representative collection from 328 winter wheat accessions	90	The experiment was conducted under rainout shelters. The control plots were provided with a total 2000 m^3^/ha of water at different growth stages while the drought stress plots were provided with only 1200 m^3^/ha.	Five genotypes were identified as highly drought.	(Chen et al., 2012)
14	Northwest Agricultural and Forestry University, Yangling, China	The wheat alien chromosome addition lines derived using Chinese Spring as the common parent, were evaluated using 10 important agronomic traits under irrigated and water-limited conditions.	Landrace derived during the advanced lines	82	The control and drought stress treatments were provided with a total of 210 mm and 120 mm irrigation, respectively, in a two year evaluation using rainout shelters.	The result showed that 26 out of 82 lines possessed high levels of drought tolerance.	(Liu et al., 2015)
15	China Agricultural University, Beijing, China Ministry of Agriculture, Xinjiang, China Xinjiang Academy of Agricultural Sciences, Xinjiang, China Chinese Academy of Sciences, Beijing, China	Two season (2011 and 2012) evaluation was performed involving wheat genotypes from the CIMMYT Wheat Physiological Germplasm Screening Nursery (CWPGSN) and seven Chinese local spring wheat genotypes. The goal was to identify the most stable genotypes across water-stressed and controlled conditions.	Local genotypes and CIMMYT advanced lines	145	Water was supplied by drip irrigation. In the no-stress condition, irrigation was supplied seven times and 8 times during 2011 and 2012, respectively. In the drought stress condition, irrigation was supplied only two and three times during 2011 and 2012, respectively.	Seven lines from CWPGSN and three local varieties (Xinchun 11, Xinchun 23 and Xinchun 29) were found to be the most stable.	(Zhang et al., 2019)
16	Bulgarian Academy of Sciences, Sofia, Bulgaria Slovak University of Agriculture, Nitra, Slovak Republic	Seedlings of six modern semi-dwarf and six old tall genotypes were evaluated for drought stress tolerance.	Modern cultivars and landraces	12	The seedlings were subjected to stress by withholding water for six days starting 14 days after planting.	The results indicated that the modern varieties were more drought-tolerant, as the water balance maintenance was better compared to the old varieties.	(Petrov et al., 2018)
17	University of Idaho, Aberdeen, USA Northwest Agricultural and Forestry University, Yangling, China Institute of Water Saving Agriculture in Arid Regions of China, Yangling, China USDA-ARS, Small Grains and Potato Germplasm Research Unit, Aberdeen, USA	Assessment of wheat genotypes for drought tolerance under irrigated and terminal drought environment was performed in the 2012–2013 and 2013–2014 seasons.	Winter wheat accessions from the USDA-ARS National Small Grains Collection	198	The terminal drought stress was imposed by stopping irrigation after heading.	Based on drought susceptibility index and membership function value of drought tolerance, 23 accessions were reported to have drought tolerance.	(Liu et al., 2017)
18	University of Faisalabad, Punjab, Pakistan University of Islamabad, Islamabad, Pakistan Yunnan Academy of Agricultural Sciences, Kunming, China	A diversity panel of bread wheat genotypes was used to assess the selection criteria for drought tolerance at the seedling stage. Seedling traits, including root length, fresh weight, dry weight cell membrane thermo-stability and chlorophyll b, were suggested to improve genetic gain for drought tolerance.	A panel of landraces, historical Pakistani varieties and advanced breeding lines	105	Greenhouse experiments were conducted by maintaining 100% and 50% of field capacity in the non-stress and drought stress treatments, respectively.	Out of these 105 genotypes, 10 drought-tolerant genotypes were identified.	(Ahmed et al., 2019)
19	Imam Khomeini International University, Qazvin, Iran Ilam University, Ilam, Iran The University of Western Australia, Perth, Australia	A panel of wild relatives of wheat were tested under irrigated and water-stressed conditions.	Wild relatives of wheat	180	A greenhouse pot experiment was conducted by applying drought stress after the three-leaf stage by subjecting treatments to 30% field capacity compared to 100% field capacity for the control.	The result showed that 12 accessions were highly superior in drought tolerance. One remarkable observation was that *Ae. speltoides* (suggested source of B genome) and *Ae*. *tauschii* (source of D genome) responded very well to drought stress.	(Ahmadi et al., 2018)
20	Agricultural Research, Education and Extension Organization (AREEO), Iran	A set of landraces, with the majority being of Iranian origin, were tested under irrigated and drought treatments.	Landraces	97	The field experiment was conducted by applying regular irrigation for the control, while irrigation was stopped at heading in the stress treatment.	Based on the drought indices used in the evaluation, superior genotypes for drought tolerance were identified.	(Arshad et al., 2016)

In addition to landraces of *T. aestivum*, other domesticated wheat species such as *T. compactum* Host, *T. sphaerococcum* Perc., *T. durum* Desf, *T. turgidum* *T. turanicum* Jakubz (“Kumut”) and *T. polonicum* L., are also sources of valuable alleles to enable breeding of wheat for drought tolerance (Janni et al., 2018; Szabo-Hever et al., 2018; Guellim et al., 2019; Nemtsev et al., 2019). Wild relatives of wheat and other related genera such as *Aegilops* are additional valued sources of germplasm for breeding drought-tolerant varieties (**Table 1**).

The rye (*Secale cereale* L.) derived wheat genotypes, also known as the 1B/1R chromosome translocation lines, have been utilized by different wheat breeding programs (Kumar et al., 2003; Hoffmann, 2008; Ren et al., 2017). These 1B/1R chromosome translocation genotypes consist of the long arm of wheat chromosome 1B including its centromere, and the short arm or a portion of rye chromosome 1R (Cai and Liu, 1989; Heslop-Harrison et al., 1990). These genotypes are also important sources of abiotic stress tolerance including drought-tolerance (Kumar et al., 2003; Rajaram, 2005). Hoffmann (2008) demonstrated that the 1B/1R translocation wheat genotypes possess greater drought tolerance. However, this may further depend on the genetic background of the recipient wheat genotype (Monneveux et al., 2003; Singh et al., 1998) as shown by Tahmasebi et al. (2015) where 1B/1R translocation genotypes were not suitable sources of diversity for improving drought tolerance.

The challenges in utilizing these landraces and wild relatives are often associated with epistatic and pleiotropic effects of some genes, leading to linkage drag (Sehgal et al., 2015). However, these unfavorable genetic effects can be minimized through recurrent backcrossing with elite genetic materials and selection. In general, the development of modern varieties has reduced the genetic diversity of bread wheat, and this reduction in diversity shows both spatial and temporal trends (Rauf et al., 2010). Studies have shown that there is extensive reduction of nucleotide diversity in the A and B genome in modern wheat, including elite lines, compared to their progenitors (Haudry et al., 2007; Dreisigacker et al., 2008). Nonetheless, elite germplasm is a convenient source of genetic variation and has been considerably utilized in breeding for drought-tolerant wheat (**Table 1**). Most of the breeding programs around the world work on elite materials to develop drought-tolerant cultivars despite the advantages that landraces and wild relatives potentially carry. The major reason behind this is that in elite materials, there is less linkage drag associated with co-inheritance of undesirable and defective genes and rare alleles. As a result, elite materials have fewer challenges related to pleiotropic and epistatic gene effects in breeding populations (Sehgal et al., 2015; Mwadzingeni et al., 2017). The advantages offered by modern elite genetic materials compared to landraces and wild relatives make enhancement of genetic gain and selection response more rapid.

As a way to move forward, synthetic hexaploid wheats (SHWs) have been developed to widen the genetic variation in modern wheat. SHWs have deployed ancestral genomes using interspecific hybridization techniques (Reynolds et al., 2007; Khan et al., 2016). The SHW lines (AABBD′D′) have been developed by artificial hybridization of tetraploid durum wheat (*T. turgidum*, AABB) and diploid wild goat grass (*Aegilops tauschiii*, D′D′) (Reynolds et al., 2007; Jafarzadeh et al., 2016; Khan et al., 2016; Li A. et al., 2018; Rosyara et al., 2019). A study involving CIMMYT SHWs has demonstrated the importance of the D′ genome of *Ae. tauschii* in maintaining genetic diversity as well as improving genetic gain (Rosyara et al., 2019). The diversity in the D′ genome may be contributing to increased stress tolerance in SHW lines (Khan et al., 2016). In one study, six SHW lines were compared to four winter wheat cultivars grown in the U.S. Great Plains under drought stress (Becker et al., 2016). One of the SHW lines was shown to be superior under drought conditions for root morphological traits including deep root biomass and length of the longest root. In another study, an evaluation of 33 *Ae. tauschii* accessions and corresponding SHW lines revealed a wide range of variability for drought tolerance (Sohail et al., 2011). CIMMYT’s efforts to develop SHW lines based on 600 genebank *Ae. tauschii* accessions has been successful, as indicated by the encouraging adoption of SHW cultivars in India and South-West China; of these, at least 30% have been reported to be drought-tolerant (Aberkane et al., 2019). Similarly, Song et al. (2017) found that six out of 34 SHW lines were drought-tolerant, and these SHW lines showed high antioxidant activities (superoxidase and peroxidase activities) that minimize drought-associated oxidative cellular damage, and thus improve drought tolerance (Zhang and Kirkham, 1994; Laxa et al., 2019). In this study, the SHWs also demonstrated superior drought tolerance over the parental lines (Song et al., 2017). Similarly, Lopes and Reynolds (2011) observed that under drought conditions, four synthetic hexaploid derived lines out-yielded the parental lines by an average of 26%. Combined, these studies suggest that there are opportunities to exploit broad genetic variation in SHWs to improve different agronomic traits including drought tolerance in bread wheat.

Furthermore, mutation breeding, including using elite germplasm, can be another avenue to create variants suitable for drought adaptation. Among the cereals, wheat is one of the major crops in which mutation breeding has been employed for cultivar development. For example, “Sharbati Sonora,” an early maturing wheat cultivar developed by gamma radiation of a Mexican cultivar, made a major contribution to wheat production in India (Raina et al., 2016). Using gamma ray radiation, 11 drought-tolerant wheat mutant lines were identified in a recent study (Sen et al., 2017). Although mutation breeding is less common compared to other breeding methods, it has potential to generate novel stress tolerance alleles.

The major sources of the above mentioned genetic resources are national and international genebanks that maintain extensive collections of wheat landraces, wild relatives, breeding populations, obsolete varieties, and modern elite varieties. The diversity of wheat germplasm deposited in these genebanks appears to be under-utilized in breeding wheat for drought tolerance, though there are growing initiatives to evaluate and identify accessions that are suitable for wheat breeding programs. These initiatives include: The Wheat Pre-breeding Project (https://www.cwrdiversity.org/partnership/wheat-pre-breeding-project/), The Wheat Improvement Strategic Programme (WISP) Consortium (http://www.wheatisp.org/Consortium/WISP.php) and Seeds of Discovery (SeeD) (https://seedsofdiscovery.org/) (Singh S. et al., 2018). Genesys (https://www.genesys-pgr.org/welcome) is an online portal that hosts information about plant genetic resources deposited in genebanks. Out of almost 320,000 wheat accessions documented in Genesys, 23% are landraces, and 7% are wild relatives. The International Maize and Wheat Improvement Centre (CIMMYT) (http://www.cimmyt.org/seed-request/) hosts the most extensive collection of wheat germplasm (102,375 accessions). USDA-ARS genebanks hold 67,615 wheat accessions: most of them are maintained at the National Small Grains Collection (https://www.ars.usda.gov/pacific-west-area/aberdeen-id/small-grains-and-potato-germplasm-research/docs/national-small-grains-collection/) in Aberdeen, ID. Wild species are held at the Wheat Genetic Resource Center (WGRC) (https://www.k-state.edu/wgrc/), and additional materials at the Germplasm Resources Information Network (GRIN) (https://www.ars-grin.gov/). The Australian Grains Genebank (http://www.seedpartnership.org.au/associates/agg) has 42,624 accessions, and the International Center for Agriculture Research in the Dry Areas (ICARDA) (http://www.icarda.org/) has 41,471 accessions. ICARDA and CIMMYT together are managing 1,570 accessions of *Ae. tauschii* (Aberkane et al., 2019). Other organizations maintaining wheat germplasm are: Navadanya in India (http://www.navdanya.org/), the Svalbard Global Seed Vault in Norway (https://www.croptrust.org/our-work/svalbard-global-seed-vault/) and the National Bureau of Plant Genetic Resources (NBPGR) in India (http://www.nbpgr.ernet.in/). However, a limitation associated with the Svalbard, Navadanya, and NBPGR collections is their restricted access.

Once genetic diversity has been identified for crossing, a double haploid (DH) strategy can be employed. The DH lines possess identical copies of chromosomes in the genome which are complete homozygotes: this allows quick fixation of desired alleles. Wheat DH populations (Fleury et al., 2010; Yan et al., 2017) show potential in improving drought tolerance, as they enhance selection efficiency by improved additive genetic variance for complex quantitative traits (Dashti et al., 2007; Mwadzingeni et al., 2017). The DH lines are considered important for QTL x environment interactions, as trait means are estimated more efficiently, resulting in precise selection due to complete homozygosity (Shamasbi et al., 2017). DH breeding, augmented by advanced molecular tools, has potential to contribute to breeding drought-tolerant wheat. Different studies in wheat have shown the potential of DH lines as sources of stress tolerance. For example, Fatima et al. (2018) identified five drought-tolerant DH lines out of 84, which were tested under control and drought stress conditions. However, DH lines are associated with negative effects from colchicine treatment, and also gametoclonal and somaclonal variation that affect plant performance. Furthermore, a high level of homozygosity may make DH lines inferior to conventionally developed inbred lines (Niemirowicz-Szczytt, 1997). Other major challenges associated with DH in breeding for drought tolerance are financial expense and unique technical expertise involving plant cell and tissue culture techniques.

## Molecular Breeding of Wheat for Drought Tolerance

### Identification of QTLs for Drought Tolerance Related Traits

By employing the above genetic diversity, conventional linkage mapping, using biparental populations and double haploid lines, has commonly been used to locate QTLs/genes associated with target traits, including those associated with drought (Sallam et al., 2016; Zhao et al, 2018; Li L. et al., 2019). The objective of these studies is to facilitate marker/genome assisted selection. As the mapping populations used in traditional linkage mapping are derived by hybridization between two parents (most cases), and have limited genetic variation, only low natural allelic diversity can be captured which results in low resolution QTLs (Sallam et al., 2016; Zhao et al., 2018). Furthermore, the large genome size of wheat, which has different epistatic interactions among the QTLs, in addition to a large number of genes influencing a trait, have reduced the identification of useful QTLs (Ashraf, 2010). However, more recently, genome-wide association studies (GWAS) studies have been used to identify marker-trait associations for the trait(s) of interest (Corvin et al., 2014; Scherer and Christensen, 2016). GWAS represents a powerful alternate to linkage mapping. GWAS facilitates the exploration of the genetic variation of complex quantitative traits such as drought, controlled by several genes and their interactions (Kooke et al., 2016). GWAS exploits linkage disequilibrium (LD) resulting from variants at a locus caused by different factors such as historical mutations, natural and artificial selection, and other forces (Wang et al., 2012; Huang and Han, 2014; Visscher et al., 2017). The method accommodates natural populations with diverse genetic backgrounds (Huang and Han, 2014) and hence takes advantage of the diversity available in the genebanks described above. The efficiency of GWAS depends upon individual factors such as the number of loci for a trait that segregates in the population, genetic architecture, and size of the study population (Visscher et al., 2017). For more precise detection of QTLs/genes, some studies have combined linkage mapping and GWAS (Sallam et al., 2016; Shi W. et al., 2017; Liu et al., 2018; Zhao et al., 2018, Li G. et al., 2019).

GWAS and linkage mapping of drought tolerance QTLs have been enabled by the availability of a high-quality reference genome for common wheat along with next-generation sequencing (NGS) technologies (Kilian and Graner, 2012; Ray and Satya, 2014; Ramirez-Gonzalez et al., 2015; IWGSC, 2018). Genotype by sequencing (GBS), a DNA sequencing method that follows the NGS protocol, does not require prior genome sequence information and has enormous potential to genotype complex genomes such as in wheat (Poland et al., 2012; Mwadzingeni et al., 2016a; Chung et al., 2017).

Using these methodologies, important QTLs for wheat physio-morphological traits associated with drought tolerance have been reported in various studies. Many earlier studies have identified genomic regions associated with different physiological traits such as carbon isotope discrimination (ΔC) on chromosomes 1BL, 2BS, 3BS, 4AS, 4BS, 5AS, 7AS, and 7BS (Rebetzke et al., 2008); flag leaf senescence on chromosomes 2B and 2D (Verma et al., 2004); coleoptile length on chromosomes 4B and 6A (Rebetzke et al., 2001); seedling vigor on chromosome 6A (Spielmeyer et al., 2007); and canopy temperature on 1B, 2B, 3B, 4A, and 5A (Pinto et al., 2010). Building upon these earlier discoveries, the last few years have shown significant advances. For example, Tura et al. (2020) mapped yield QTLs under drought in a double haploid population on chromosomes 4A, 5B, and 7A. In a comprehensive GWAS study involving a panel of 210 European elite wheat lines, Touzy et al. (2019) discovered 24, 31, and 28 QTLs associated with drought tolerance, respectively, under low, medium and high water stress conditions. Bhatta et al. (2018) identified 90 marker-trait associations (MTAs) related to yield and associated traits under limited water conditions in a GWAS study with GBS markers using 123 synthetic hexaploid wheat lines. Similarly, QTLs for drought-related traits such as the drought susceptibility index (DSI), normalized difference vegetative index (NDVI), and leaf traits (including green leaf area, leaf senescence, and flag leaf phenotypes) were detected on chromosomes 1B, 4A, 6B, 5B, 7A, and 7B (Edae et al., 2014) in a GWAS involving a spring wheat panel. **Table 2** summarizes QTLs reported for drought tolerance related traits on different chromosomes of the wheat genome, published in the peer reviewed literature from 2011 onwards.

**Table 2:** Recent peer reviewed reports (2011-current) of the important physio-morphological traits associated with drought tolerance and locations of QTLs associated with these traits in wheat.

Target trait	Chromosome	Method used for QTL identification	Type of population	Environment	Reference
Days to heading	1A, 2A, 2B, 3A, 4B, 5B, and 6D	GWAS	Diversity panel	Rainfed	Gahlaut et al., 2019
	1B, 2D, 3B, and 4A	GWAS	Diversity panel	Drought stress	Mathew et al., 2019
	2A	GWAS	Diversity panel	Drought stress	Qaseem et al., 2018
	2D and 6A	Linkage mapping	Double haploid population	Drought stress at flowering	Fatima et al., 2018
Days maturity	1A, 1B, 2A, and 4B	GWAS	Diversity panel	Rainfed	Gahlaut et al., 2019
	4B and 7A	GWAS	Elite wheat genotypes	Drought stress	Qaseem et al., 2019
	2B, 5A, and 6B	GWAS	Diversity panel	Drought stress	Mathew et al., 2019
	7A	GWAS	Diversity panel	Drought stress	Qaseem et al., 2018
	1B	Linkage mapping	Double haploid population	Drought stress at flowering	Fatima et al., 2018
Grain yield	4A, 3B, and 7A	Linkage mapping	Doubled haploid population	Rainfed	Gahlaut et al., 2017
	1A, 1B, 2D, and 3B	Linkage mapping	RIL population	Terminal drought stress	Zandipour et al., 2020
	1D, 3D, and 5B	GWAS	Diversity panel of 94 German winter wheat	Drought stress	Lehnert et al., 2018
	2B, 6D	GWAS	Diversity panel	Rainfed	Li L. et al., 2019
	3D, 6A, 6D, and 7B	GWAS	Elite wheat genotypes	Drought stress	Qaseem et al., 2019
	6D	Linkage mapping	Synthetic derived RIL population	Drought stress	Liu et al., 2019
	2A and 4A	GWAS	Diversity panel	Drought stress	Mathew et al., 2019
	2A and 7A	GWAS	Diversity panel	Drought stress	Qaseem et al., 2018
	2D	GWAS	Wheat association mapping initiative (WAMI) panel	Drought stress	Lopes et al., 2015b
	1AL	GWAS	Spring wheat diversity panel	Rainfed	Ain et al., 2015
	2B, 3B, 4A, 5D, 6B, and 7A	GWAS	Diversity panel	Irrigated	Gahlaut et al., 2019
Yield stability index	1B, 2A, 2B, 3A, 3B, 5A, 5D, 6A, and 6D	GWAS	Diversity panel	Rainfed and irrigated	Ballesta et al., 2020
Harvest index	7B and 7D	GWAS	Elite wheat genotypes	Drought stress	Qaseem et al., 2019
	1B, 3B, 4A, 5B, and 6B	GWAS	Diversity panel	Drought stress	Lehnert et al., 2018
Spike length	1B, 2B, 2D, 3A, 4B, 5B, 6A, 6B, and 7A	GWAS	Diversity panel	Drought stress	Mwadzingeni et al., 2017
	6A	Linkage mapping	Double haploid population	Drought stress at flowering	Fatima et al., 2018
	4B and 6B	GWAS	Elite wheat genotypes	Drought stress	Qaseem et al., 2019
Spikelet per spike	6B, 2D, 2B, 5D, 1B, and 4B	GWAS	Diversity panel	Drought stress	Mwadzingeni et al., 2017
	2B and 4B	GWAS	Elite wheat genotypes	Drought stress	Qaseem et al., 2019
Number of kernels per spike	2D and 4A	GWAS	Diversity panel	Drought stress	Mwadzingeni et al., 2017
	6A, 7A, 1B, 3B3, 3B, 6B2, 7B, and 1D	Linkage mapping	RIL population	Terminal drought stress	Zandipour et al., 2020
	2A	Linkage mapping	Doubled haploid population	Osmotic stress	Dolferus et al., 2019
	5A	Linkage mapping	Doubled haploid population	No osmotic stress	Dolferus et al., 2019
	1A, 2B, 3D, 4A, and 4B	GWAS	Diversity panel	Rainfed	Li L. et al., 2019
	2A	Linkage mapping	Synthetic derived RIL population	Drought stress	Liu et al., 2019
	6A and 7B	Linkage mapping	Double haploid population	Drought stress at flowering	Fatima et al., 2018
Thousand grain weight	7A	Linkage mapping	Doubled haploid population	Rainfed	Gahlaut et al., 2017
	6D	GWAS	Diversity panel	Drought stress	Lehnert et al., 2018
	7A	Linkage mapping	Synthetic derived RIL population	Drought stress	Liu et al., 2019
	2D and 6A	Linkage mapping	Double haploid population	Drought stress at flowering	Fatima et al., 2018
	1B, 2B1, 2B2, and 3B	Linkage mapping	RIL population	Terminal drought stress	Zandipour et al., 2020
	2A, 5A, and 6B	GWAS	Diversity panel	Rainfed	Li L. et al., 2019
	2A	Linkage mapping	Synthetic derived RIL population	Drought stress	Liu et al., 2019
	5A	Linkage mapping	Double haploid population	Drought stress at flowering	Fatima et al., 2018
	1B, 2D, 3A, 3B, 3D, 4D, 5A, 6A, 6B, 7A, and 7B	Linkage mapping	Doubled haploid population	Rainfed and irrigated	Shi S. et al., 2017
	2A	Linkage mapping	Core collection	Rainfed and irrigated	Ahmad et al., 2014
	2A, 2D, 3A, 4A, 4B, 5B, and 6A	GWAS	Diversity panel	Rainfed	Gahlaut et al., 2019
	1AL, 4AL, 7AS, 7DS	Linkage mapping	F_2_:F_3_ population	Drought stressed	Nezhad et al., 2012
Grain weight per plant	2D	GWAS	Diversity panel	Drought stress	Schmidt et al., 2020
Awn length	1D, 2A, 2B, 3B, 4A, 4B, 4D, 5A, 5B, 5D, 6B, and 7A	GWAS	Synthetic hexaploid wheat	Drought stress	Bhatta et al., 2018
	7D	GWAS	Diversity panel	Drought stress	Qaseem et al., 2018
	4B and 4D	GWAS	Diversity panel	Drought stress	Schmidt et al., 2020
	2A	GWAS	Diversity panel	Drought stress	Qaseem et al., 2018
	5BL and 6AL	GWAS	Diversity panel	Rainfed	Ain et al., 2015
	5A	Linkage mapping	Double haploid population	Rainfed	Gahlaut et al., 2017
	1A and 2D	GWAS	Diversity panel	Drought stress	Mwadzingeni et al., 2017
	4B	GWAS	Wheat association mapping initiative (WAMI) panel	Drought stress	Lopes et al., 2015b
	4B, 6B	GWAS	Diversity panel	Irrigated	Gahlaut et al., 2019
	1B, 3B, 5A	GWAS	Diversity panel	Rainfed	Gahlaut et al., 2019
	7B and 7D	GWAS	Elite wheat genotypes	Drought stress	Qaseem et al., 2019
Tillers per plant	1B, 2B, 4B, 7B	GWAS	Elite wheat genotypes	Drought stress	Qaseem et al., 2019
Chlorophyll content	1B, 2A, 2B, 2D, 3A, 3B, 4B, 4D, 5A, 6B, 6D, and 7A	Linkage mapping	Double haploid population	Rainfed and irrigated	Shi S. et al., 2017
Normalized difference vegetative index (NDVI)	1A, 1B, 1D, 2A, 2B, 2D, 3A, 3B, 3D, 4A, 4B, 4D, 5A, 6A, 6B, 6D, 7A, and 7B	Linkage mapping	Double haploid population	Rainfed and irrigated	Shi S. et al., 2017
	2A	Linkage mapping	Synthetic derived RIL population	Drought stress	Liu et al., 2019
	6D	Linkage mapping	Synthetic derived RIL population	Drought stress	Liu et al., 2019
Flag leaf senescence	1B, 2B, and 3B	Linkage mapping	BC_2_F_2_ families	Drought stress	Barakat et al., 2015
	1B, 4A, 6B, 5B, 7A, and 7B	GWAS	Diversity panel	Drought stress	Edae et al., 2014
Flag leaf length	7B and 7D	GWAS	Elite wheat genotypes	Drought stress	Qaseem et al., 2019
Flag leaf width	4B and 6A	GWAS	Elite wheat genotypes	Drought stress	Qaseem et al., 2019
Leaf area	7A, 7B, and 7D	GWAS	Elite wheat genotypes	Drought stress	Qaseem et al., 2019
Germination ability	5A, 3B, 4B, and 6B	Linkage mapping	Mapping population	Drought stress	Czyczyło-Mysza et al., 2014
Coleoptile length	5D	Linkage mapping	RIL population	Osmotic stress	Zhang et al., 2013
Seedling height	2D	Linkage mapping	RIL population	Osmotic stress	Zhang et al., 2013
Shoot biomass	1B, 2B, 2D, 3B, 5B, 7A, and 7D	GWAS	Diversity panel	Drought stress	Mathew et al., 2019
Root biomass	1B, 1D, 2B, 3B, 4A, and 4D	GWAS	Diversity panel	Drought stress	Mathew et al., 2019
Root dry biomass	3A, 3B, 3D	GWAS	Diversity panel	Drought stress	Lehnert et al., 2018
Root length	4A	Linkage mapping	RIL population	Osmotic stress	Zhang et al., 2013
	2B and 3B	GWAS	Core winter wheat collection	PEG induced water stress	Ayalew et al., 2018
	3B, 4A, and 5B	GWAS	Core winter wheat collection	Non-stress	Ayalew et al., 2018
	5B and 5D	Linkage mapping	Mapping population	Drought stress	Czyczyło-Mysza et al., 2014
	2D	GWAS	A population of elite wheat varieties	Drought stress	Ahmad et al., 2017
Root to shoot dry weight ratio	2D, 5A, and 3B	Linkage mapping	RIL population	Irrigated and Osmotic stress	Zhang et al., 2013
	2B, 4A, 4D, and 6A	GWAS	Diversity panel	Drought stress	Mathew et al., 2019
Photosynthetic rate	3A	Linkage mapping	F_2_ population	Drought stress	Maqsood et al., 2017
Relative water content	4D	Linkage mapping	F_2_ population	Drought stress	Maqsood et al., 2017
Cell membrane thermostability	2B	Linkage mapping	F_2_ population	Drought stress	Maqsood et al., 2017
	2B and 6B	Linkage mapping	BC_2_F_2_ families	Drought stress	Barakat et al., 2015
Abscisic acid content	2B, 3B, 5B, 6B, and3D	Linkage mapping	BC_2_F_2_ families	Drought stress	Barakat et al., 2015
Waxiness	2A	Linkage mapping	Synthetic derived RIL population	Drought stress	Liu et al., 2019
Low excised-leaf water loss (ELWL)	3A, 3B, 4B, 5B, 5D, 6B, 7A, 7B, and 7D	Linkage mapping	Double haploid hexaploid wheat	Irrigated	Czyczyło-Mysza et al., 2018
Above ground dry weight	7B and 7D	GWAS	Elite wheat genotypes	Drought stress	Qaseem et al., 2019
Above ground biomass	1D, 2A, and 4A,	GWAS	Diversity panel of 94 German winter wheat	Drought stress	Lehnert et al., 2018

A number of the QTLs reported in **Table 2** that are associated with different physio-morphological traits that contribute to wheat grain yield under drought, appear to be restricted to specific genetic backgrounds and environments (Griffiths et al., 2012; Acuna-Galindo et al., 2015). To determine which QTLs are relevant across genetic backgrounds and environments, some studies have used a meta-analysis approach to identify common QTLs discovered in different studies. For example, in a QTL meta-analysis recently conducted by Soriano and Alvaro (2019), 634 QTLs retrieved from earlier studies were projected on a consensus map having 7352 markers, which resulted in 94 consensus QTL regions, of which 35 were associated with root architecture and response to moisture stress. Similarly, in another QTL meta-analysis, Acuna-Galindo et al. (2015) reported 43 meta-QTLs related to drought and heat stress of which 20 were specific to drought stress tolerance. These results suggest that QTL meta-analysis, along with better estimations of QTL effects, may maximize the benefits of past drought QTL studies (Acuna-Galindo et al., 2015; Gupta et al., 2017; Soriano and Alvaro (2019).

Once candidate genes or loci have been identified, another approach is to use allele-specific markers to identify favorable alleles (haplotypes) that promote drought tolerance in a defined population. For example, Khalid et al. (2019) in a study comprising 213 advanced lines derived from synthetic hexaploid wheat and elite cultivars (CIMMYT and Pakistan), identified favorable alleles at five candidate genes associated with drought adaptation, including those that encode a cell wall invertase gene (*TaCwi-A1*) that converts sucrose into glucose and fructose, a dehydration responsive element binding protein (*Dreb1*) and *COMT-3B* that promotes lignin under water stress. The cell wall invertase gene discovery was consistent with an earlier study involving 348 modern Chinese cultivars which identified a favorable allele (haplotype) at another invertase gene (*TaCWI-5D*) on chromosome 5D in terms of promoting drought tolerance in wheat (Jiang et al., 2015).

Combined, these advances in genome sequencing, QTL and favorable allele identification, have provided wheat breeders with numerous targets for introgression and selection of drought tolerance promoting alleles that are potentially stable across environments and genetic backgrounds.

### Genome Engineering Techniques

Genome engineering techniques, including gene pyramiding, gene stacking, and transgenics, broaden the methods available to breed wheat for tolerance to abiotic stress including drought (Budak et al., 2015; Mwadzingeni et al., 2016a). Clustered Regulatory Interspaced Short Palindromic Repeats (CRISPR) is a bacterium-derived genome editing system; in this system, a DNA fragment encoding a non-coding RNA sequence is designed to target and then cleave a chromosomal DNA target of interest, within the Cas9 protein complex (Garneau et al., 2010; Budak et al., 2015; Zhang et al., 2018; Kumar et al., 2019; Okada et al., 2019; El-Mounadi et al., 2020; Shinwari et al., 2020). Recently, Wang et al. (2018) used CRISPR/Cas9 to increase grain size in wheat. Kim et al. (2018) also successfully established a CRISPR/Cas9 genome editing system in wheat protoplasts to edit the stress-responsive factor genes *TaDREB2* and *TaERF3.* Since some essential regulatory genes that control the biosynthesis of metabolites associated with drought tolerance have been identified (Yang et al., 2010), the CRISPR/Cas9 gene editing technology could potentially be used to target such genes and traits in the future (Singh B. et al., 2018).

In addition, CRISPR/Cas9 mediated base editing (Zong et al., 2017; Li C. et al., 2018) and prime editing (Anzalone et al., 2019) techniques could be potentially utilized in breeding for drought tolerant wheat in the future as alternatives to the standard CRISPR/Cas9 genome editing system. Gene editing using CRISPR technologies, which depends on double strand breaks induced by the Cas9 protein (Garneau et al., 2010), has limitations associated with the delivery of donor DNA to the targeted cell types due to the low frequency of homologous recombination in plants (Molla and Yang, 2019; Hassan et al., 2020). The advantage of base editing technology over the standard CRISPR technology is: it does not require double stranded breaks and can simply perform substitution mutations allowing four kinds of modifications (C/G-to-T/A and A/T-to-G/C) (Komor et al., 2016; Gaudelli et al., 2017; Li C. et al., 2018; Molla and Yang, 2019). However, as base editing does not create indels (Komor et al., 2016; Gaudelli et al., 2017), an alternative prime editing technology which also does not require double stranded DNA cleavage, has been engineered to introduce indels; it also allows all 12 base to base conversions (Anzalone et al., 2019). As the prime editing method overcomes the above-stated limitations and enables different mutations (point mutations and indels) in wheat protoplasts (Lin et al., 2020), it may be a game changer technology in the future in terms of engineering drought tolerant wheat (Hassan et al., 2020; Marzec and Hensel, 2020).

In addition to gene editing, there is evidence suggesting that wheat genetic improvement is also possible through genetic engineering. For instance, transfer into wheat of the *AISAP* gene encoding a stress-associated protein from the halophyte grass/Mediterranean saltgrass (*Aeluropus littoralis*) has been shown to enhance the germination rate, biomass, and grain yield of wheat under osmotic- and salinity stress (Ben-Saad et al., 2012). A recent study (Ayadi et al., 2019) demonstrated that overexpression of the wheat aquaporin gene *TdPIP2* in transgenic wheat improved drought tolerance. Similarly, the overexpression of the *Escherichia coli* *CspA* gene (modified as *SeCspA*) in transgenic wheat lowered the rate of water loss and maintained higher chlorophyll and proline under drought compared to non-transgenic plants (Yu et al., 2017). Additionally, improved drought tolerance (enhanced growth, delayed senescence, higher relative water content, higher photosynthetic rate, and higher total chlorophyll content) was observed in transgenic wheat in which an *Arabidopsis thaliana* cysteine protease (OVERLY TOLERANT TO SALT-1, *OTS1*) was over-expressed (using a ubiquitin promoter) compared to non-transformed wheat plants (le Roux et al., 2019). It has also been reported that overexpression of a fructan exohydrolase encoding gene (*1-FEH w3*) promotes higher fructan remobilization under drought which contributes positively to grain yield (Zhang et al., 2015; Hou et al., 2018). Several other studies have shown that overexpression of different drought responsive genes confers greater drought tolerance in transgenic wheat including the genes *AtHDG11* (Li et al., 2016), *TaCIPK23* (Cui et al., 2018), *TaBZR2* (Cui et al., 2019), *TaWRKY2* (Gao et al., 2018), *TaWRKY1*, and *TaWRKY33* (He et al., 2016), and *TaPYL4* (Mega et al., 2019). Interestingly, *TaSnRK2.9*, which is a sucrose non-fermenting 1-related protein kinase gene cloned from bread wheat, elevated ABA content in tobacco (*Nicotiana tabacum*) resulting in greater drought tolerance (Feng et al., 2019).

Therefore, both gene editing and genetic engineering show promise in terms of achieving drought tolerance in wheat. However, time, effort, skill, financial resources, regulatory issues, and public acceptance are major constraints to wider adoption of these techniques in wheat research programs.

### Future Perspectives

The world is currently facing the prospect of food insecurity due to increasing drought. An assessment of genetic gain over fifty years of wheat breeding at CIMMYT showed an increase in grain yield by ~18 kg/ha per year under drought (Mondal et al., 2020) but this rate will be insufficient to meet demand. As this review has shown, promising approaches to improve drought tolerance in wheat include exploration of wheat diversity in genebanks, identification of genome-wide QTLs, validation of putative QTLs, and introgression of markers, genomic segments or candidate genes. However, undertaking this pipeline *de novo* is a major challenge in terms of time, skill, and resources. Therefore, the QTL meta-analysis approach (Acuna-Galindo et al., 2015; Gupta et al., 2017; Soriano and Alvaro, 2019) could be an immediate option that utilizes the results of already-existing QTLs associated with drought tolerance in wheat. Another promising near-term technique is genome editing using CRISPR/Cas9, given the potential it has shown to improve traits such as male sterility, grain weight, protein content, and powdery mildew resistance (Zhang Y. et al., 2017; Wang et al., 2018; Zhang et al., 2018; Okada et al., 2019). Furthermore, the pioneering CRISPR-based technologies including base editing and prime editing may have potential future. Though transgenic wheat offers potential, public acceptance may limit its adoption. By contrast, synthetic hexaploid wheats (SHWs) have shown promise in terms of drought tolerance and simplify the use of existing wild genetic resources (Lopes and Reynolds, 2011; Sohail et al., 2011; Becker et al., 2016; Aberkane et al., 2019; Laxa et al., 2019).

Apart from the use of wider germplasm, the growth environment in which selection and breeding is undertaken is important as well as the phenotyping strategies employed. The tradition of breeding for drought tolerance in wheat has been to compare grain yield in a water deficit environment compared to an optimal environment (Khakwani et al., 2011). However, the complex nature of drought (e.g., drought on sandy soil is more severe than on clay soil) complicates these efforts in terms of their actual impact on yield stability (Hoover et al., 2018). Furthermore, the large and complex nature of the wheat genome makes breeding for drought-tolerant wheat challenging. Therefore, adoption of a comprehensive strategy to develop drought-tolerant wheat varieties is needed. Such a strategy requires precise phenotyping in a water deficit environment (Fleury et al., 2010) as the efficiency of genomics also rests on advances in phenomics (Araus and Cairns, 2014; Fahlgren et al., 2015; Bai et al., 2016; Fernandez et al., 2017; Zhang X. et al., 2017). Many ground-based and aerial phenotyping platforms have been developed recently, with advances made in remote sensing, aeronautics, and computing (White et al., 2012; Araus and Cairns, 2014). In addition, new bioinformatics platforms have shown potential to overcome challenges associated with the management of the high volume of data generated by precision phenotyping tools (Shi et al., 2016; Singh et al., 2016; Coppens et al., 2017; Araus et al., 2018; Yang et al., 2020). These include Minimum Information About a Plant Phenotyping Experiment (MIAPPE), a platform that enables harmonization of phenotyping experiments (Bolger et al., 2019); and Crop Ontology, a platform that promotes proper use of genotypic and phenotypic data through data annotation (Shrestha et al., 2012).

Global agricultural research institutions such as CIMMYT and ICARDA have already initiated precision phenotyping coupled with NGS protocols to improve the utilization of wheat genebank materials and their development into pre-breeding materials for stress tolerance breeding programs (Crossa et al., 2016; Singh S. et al., 2018; Singh et al., 2019). However, other national and international institutions holding wheat genetic materials may need to prioritize similar efforts in order to deploy potential germplasm into wheat breeding programs. Traditional efforts may be revived to generate new allelic diversity for drought tolerance traits through mutagenesis involving chemical mutagens, ultraviolet light, and high energy radiation (Chen et al., 2014). Similarly, to discover the genetic basis of complex traits, transcriptomics, proteomics, and metabolomics offer promise (Reddy et al., 2014). For example, the information generated concerning different metabolic pathways have potential to add value in terms of developing targeted strategies that modulate the expression of genes associated with stress tolerance (Yang et al., 2010).

Further enhancement and strengthening of current global research collaborations is another way forward. CIMMYT is using the concept of 12 mega environments (ME) (http://wheatatlas.org/megaenvironments), defined as broad geographic regions with similar biotic and abiotic stresses, as well as agronomic practices and consumer preferences, to develop and promote wheat cultivars for wider adoption. One immediate activity of this collaboration could be to support the initiation of genomic characterization of genebank materials in developing countries. In the same vein, a very promising global initiative is the “The Heat and Drought Wheat Improvement Consortium (HeDWIC)” (https://www.hedwic.org), which is a worldwide platform of wheat scientists from more than 90 countries working on drought and heat tolerance. Similarly, “The International Wheat Yield Partnership (IWYP)” (https://iwyp.org) is a recent global consortium of partners including public and private research organizations, that aims to improve the genetic potential of wheat grain yield by 50% throught collaborative efforts in two decades. It is hoped that such global scientific cooperation in wheat breeding has the potential to generate new drought-tolerant varieties to combat climate change.

## Author Contributions

KK and AN conceptualized the manuscript, while KK undertook the literature review, analysis, and wrote the manuscript, and AN and MR edited the manuscript.

## Funding

The manuscript is a part of PhD thesis (KK) supported by a grant to MR from the Canadian International Food Security Research Fund (CIFSRF), jointly funded by Global Affairs Canada and the International Development Research Centre (IDRC, Ottawa). The PhD program is also partially supported by grants to AN by the Agricultural Adaptation Council of Canada; Grain Farmers of Ontario, Canada; and SeCan, Canada.

## Conflict of Interest

The authors declare that the research was conducted in the absence of any commercial or financial relationships that could be construed as a potential conflict of interest.
